# Fibrin-Associated Large B-Cell Lymphoma of Prosthetic Aortic Valve and Aortic Root Graft

**DOI:** 10.3390/hematolrep18010001

**Published:** 2025-12-22

**Authors:** Ashish Rajput, Abdulrahim Alabdulsalam, Claribeth Ruano, Sabin J. Bozso, Anthea Peters, Michael C. Moon, Jean Deschênes

**Affiliations:** 1 Department of Laboratory Medicine and Pathology, University of Alberta, Edmonton, AB T6G 1C9, Canada; ashish.rajput@easternhealth.ca (A.R.); claribeth.ruano@saskhealthauthority.ca (C.R.); 2 Division of Cardiac Surgery, University of Alberta, Edmonton, AB T6G 2B7, Canada; bozso@ualberta.ca (S.J.B.); mmoon@ualberta.ca (M.C.M.); 3 Division of Hematology, University of Alberta, Edmonton, AB T6G 2B7, Canada; anthea1@ualberta.ca

**Keywords:** fibrin-associated large B-cell lymphoma, bioprosthetic valve, primary cardiac lymphoma, R-CEOP therapy

## Abstract

**Background and clinical significance:** Primary cardiac diffuse large B-cell lymphoma (DLBCL) arising in bioprosthetic valves is exceedingly rare. Most patients present with localized disease often masquerading as suspected thrombi or vegetations. Imaging studies are inconclusive and due to the rarity of the disease, treatment and follow-up data are very limited. **Case presentation:** We present one such case developing 9 years after aortic valve replacement in an otherwise immunocompetent patient, who presented with minor symptoms despite significant disease burden. This tumor contained Epstein–Barr virus (EBV), was confined to the site of origin, and has behaved non-aggressively after excision with a follow-up of 59 months. **Conclusions:** This unique disease is classified as Fibrin-associated large B-cell lymphoma (FA-LBCL) in view of its distinct clinical-pathological features. This report also addresses the unique features of this type of lymphoma.

## 1. Introduction

Fibrin-associated large B-cell lymphoma (FA-LBCL) of cardiac prosthetic valve is a recently described entity with long evolution and good prognosis. Only seven cases involving lymphoma of cardiac prosthetic valves have been reported with limited follow-up data [1,2,3,4]. Imaging studies mimic the findings of thrombus or vegetations, and hence lymphoma is often not suspected on initial evaluation. The disease is often localized, and patients are usually immunocompetent with no previous history of lymphoma. Patients can present insidiously or can have a fulminant course, as in our case. There is no clear consensus on the exact pathologic classification as well as treatment guidelines. The histopathological diagnosis has recently been updated as these cases do not fit in the broad category of DLBCL. This case report reviews all the other cases reported in the literature, classifies the lesion accurately as FA-LBCL, and proposes systemic chemotherapy in these patients with localized diseases [5,6,7].

## 2. Case Report

A 40-year-old immunocompetent male patient presented with complaints of dizziness. Further investigation revealed an 8 cm ascending aorta and aortic root aneurysm resulting in severe aortic regurgitation. He therefore underwent an ascending aortic replacement using a 28 mm Gelweave prosthetic graft and a Bentall procedure using a 29 mm Freestyle bioprosthetic aortic valve. He was discharged from hospital without complication.

**Figure 1:**
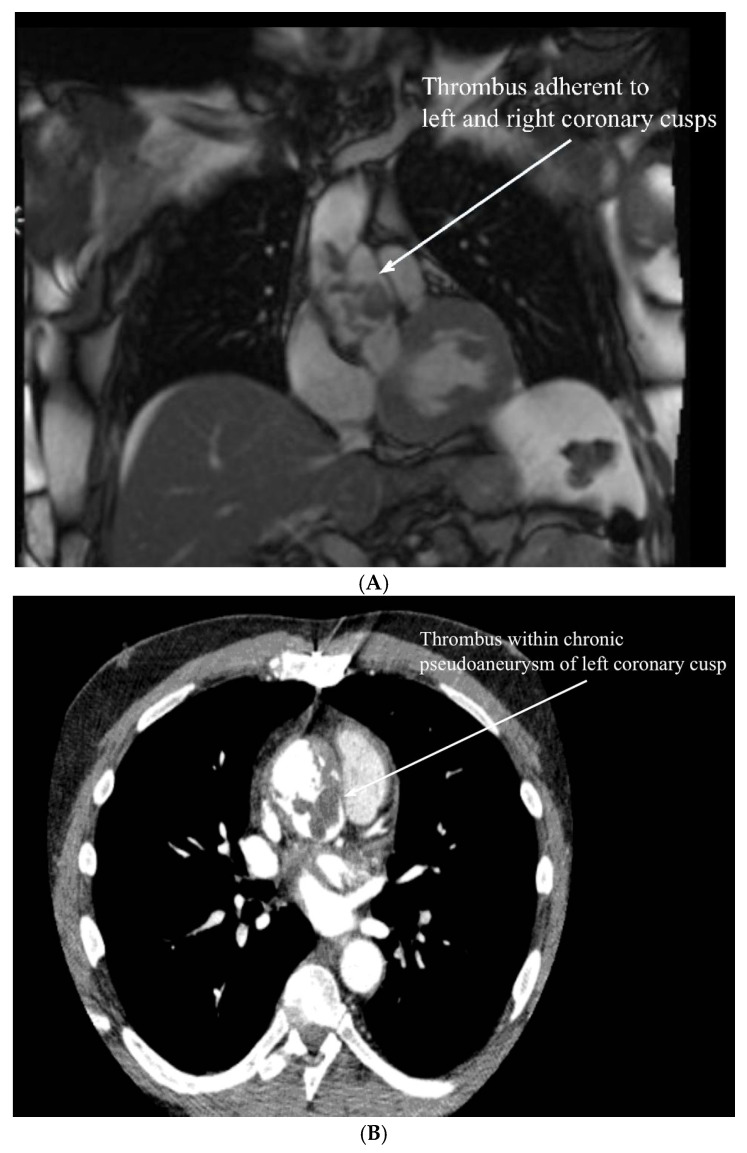
(**A**). MRI showing a thrombus extending from the aortic valve leaflets up to the level of the distal anastomosis of the interposition aortic graft. (**B**). CT scan showing thrombus within pseudoaneurysm of left coronary cusp.

He presented nine years thereafter with shortness of breath and decreased exercise tolerance. There were no night sweats, fever, or weight loss. A transthoracic echocardiogram demonstrated normal biventricular function with thickening and immobility of the non-coronary cusp of the bioprosthetic aortic valve causing mild aortic regurgitation. A magnetic resonance imaging (MRI) scan demonstrated a pseudoaneurysm of the left coronary button with suspected contained rupture. There was no suspicion of lymphoma. A large amount of presumed thrombus material extended from the aortic valve leaflets up to the level of the distal anastomosis of the interposition aortic graft (Figure 1A).

Computed tomography (CT) angiogram showed extravasation of the dye and an aneurysmal aortic root (Figure 1B).

Immediate surgery was performed, using a Cabrol procedure with an 8 mm Gelweave graft.

EBV serologic testing with anti-VCA IgM and IgG as well as anti-EBNA-1 IgG showed positive results that suggested late primary infection or EBV reactivation in someone who was remotely infected. The aortic valve thrombus tissue culture showed no growth.

**Figure 2:**
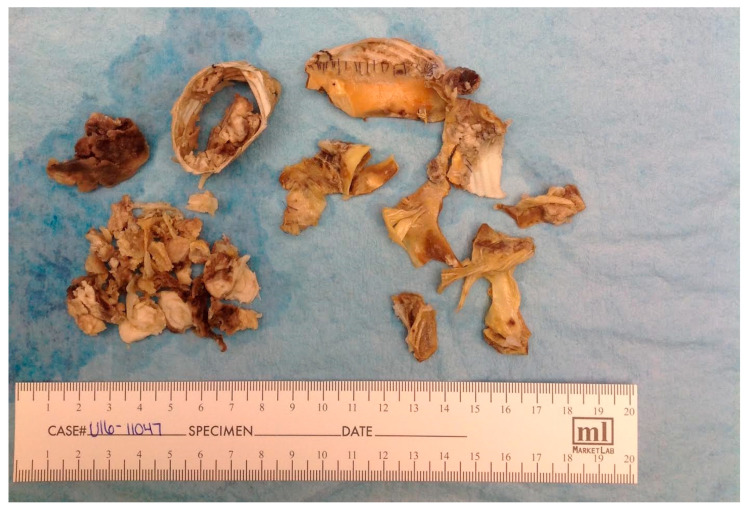
Gross photograph of the excised prosthetic aortic root and valve leaflets.

Macroscopically, a portion of heavily calcified aorta and aortic valve leaflet prosthesis, with attached thrombi, were received (Figure 2).

**Figure 3:**
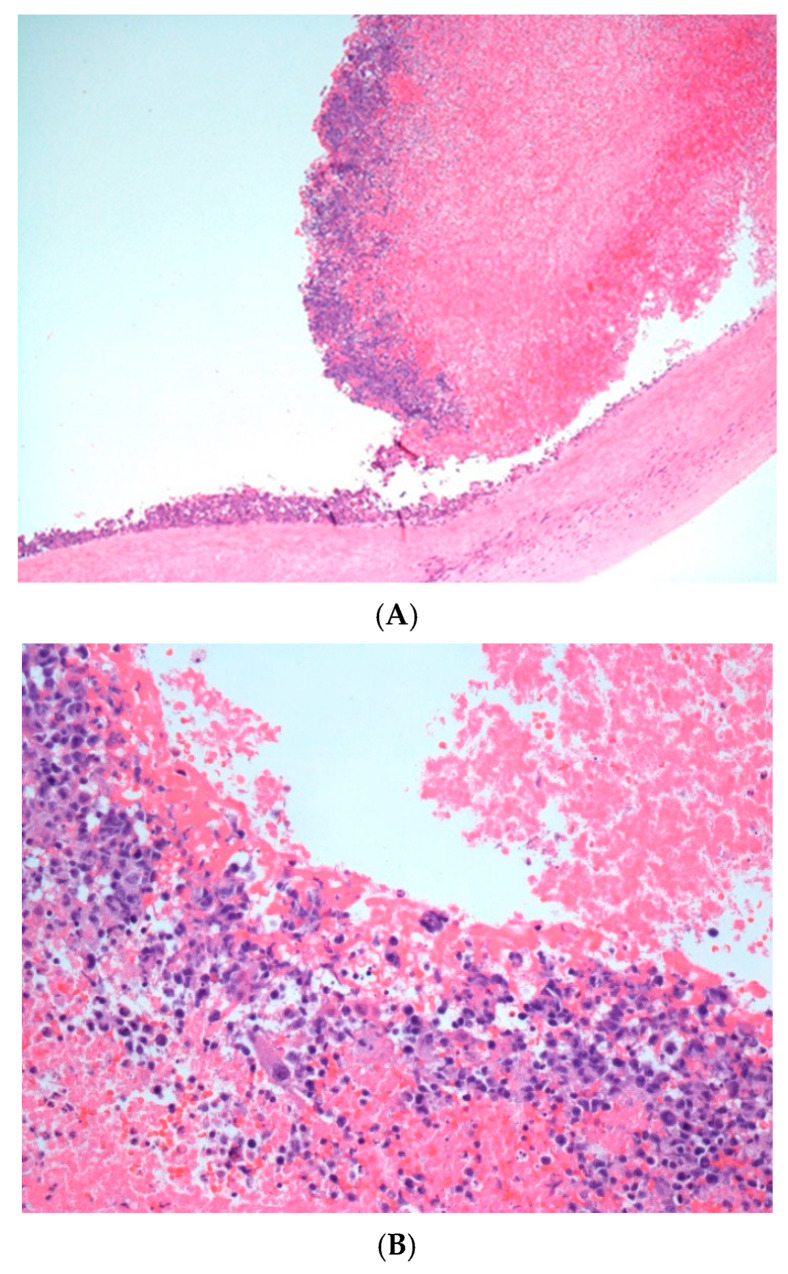
(**A**)**.** Superficial thrombotic material containing lymphoid cellular aggregates (Olympus microscope, 4×). (**B**). Luminal surface of explanted valve showing atypical lymphoid infiltrate composed of moderate to large-sized, pleomorphic cells with apoptosis, mitosis, and necrotic areas (Olympus microscope 20×).

Microscopically, both the valves and aorta contained attached thrombotic fibrinoid material that contained aggregates of large atypical lymphoid cells, associated with apoptotic and mitotic activity. These cells were confined to the surface of the thrombi with no deep extension into underlying tissue (Figure 3A,B).

**Figure 4:**
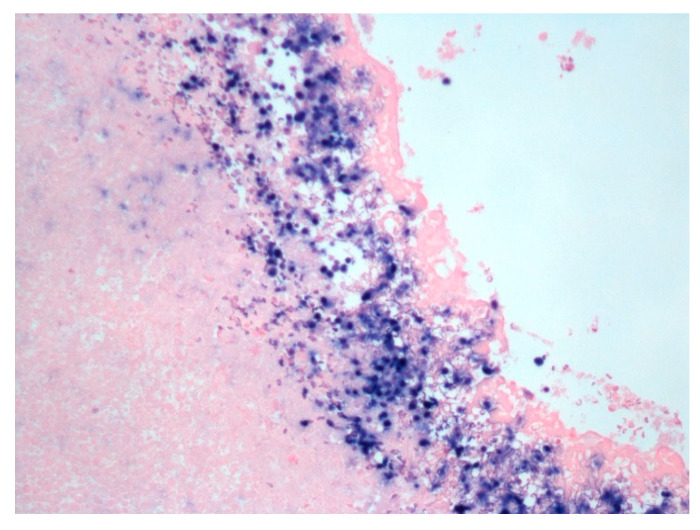
Epstein–Barr virus-encoded RNA in situ hybridization for EBV shows diffuse nuclear staining in the malignant cells (Olympus microscope, 20×).

EBV-encoded RNA (EBER) in situ hybridization showed diffuse positive staining (Figure 4).

**Figure 5:**
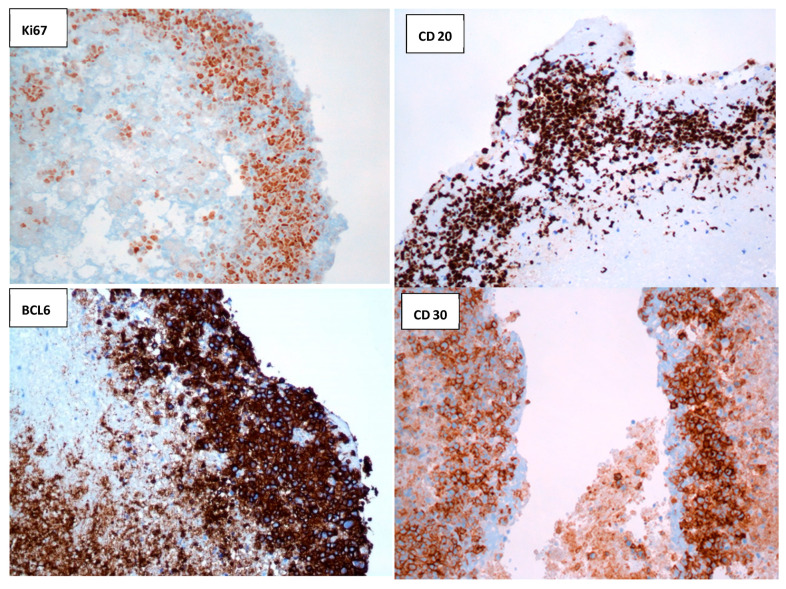
Immunohistochemical studies: the malignant cells stained positively with antibodies directed against Ki-67, CD20, BCL6, and CD30, favors the diagnosis of malignant B-cell lymphoma (Olympus microscope, 20×). T-Cell markers CD3 and CD5 were negative.

Immunohistochemical studies showed the atypical cells to be positive for CD20, PAX5, CD30, BCL2, and BCL6 and negative for CD10, CD3, CD5, and ALK1, with equivocal staining for MUM1 (Figure 5).

Ki-67 proliferation index was estimated at 95%. A diagnosis of EBV-associated Diffuse Large B-Cell Lymphoma (DLBCL) arising in a bioprosthetic valve was made.

The patient proceeded to staging workups by imaging studies which showed no other sites of disease. Positron emission tomography scanning showed no evidence of local residual disease. Due to the unusual location of the lymphoma and difficulty in assessing the extent of involvement, it was decided to give the patient four cycles of systemic R-CEOP (Rituximab—cyclophosphamide, epirubicin, vincristine, and prednisone) chemotherapy. The therapy was initiated five months after the surgery. The patient has finished all four cycles of chemotherapy without any adverse effects and was stable at 59-month interval post therapy.

## 3. Discussion

Primary cardiac lymphoma (PCL) is a rare disease. It accounts for 0.5% of all extra nodal lymphomas [1]. Most cases of lymphoma involving the heart are secondary to lymphoma elsewhere in the body. The largest study of PCL in the literature reported 197 cases, and the most commonly reported subtype is DLBCL [2]. In general, PCL has a poor outcome with median overall survival of approximately 12 months.

Most reported cases of PCL involve heart chambers with infiltration of myocardium. Very few cases primarily involve heart valves. In recent years, several case reports of PCL of DLBCL type arising in association with prosthetic heart valves and aortic root grafts have been reported [3,4]. We found seven cases involving prosthetic heart valves, and those are presented in a recent article by Farah et al. [4]. These cases tend to present with a relatively long interval after the graft procedure and are in the majority EBV-positive. Interestingly, these cases typically present with localized disease often masquerading as suspected thrombi or vegetations, which, on histologic examination, contain lymphoma cells embedded in an acellular fibrinous material, with no extension to underlying tissue. The lymphoid proliferation does not show evidence of systemic spread, and death from systemic lymphoma does not occur, regardless of whether they received therapy or not. However, the case described by Farah et al. recurred in less than a year and the patient was not given chemotherapy. Given their association with a foreign implanted device and the relatively long interval between disease presentation and the initial surgical graft implantation procedure, these lymphomas have been linked to the broader category of DLBCL associated with chronic inflammation (DLBCL-CI), as described in the most recent 2022 WHO classification [5]. DLBCL-CI occurs in association with chronic longstanding infection (e.g., pyothorax-associated lymphoma) or with various types of implants and prostheses. They are often aggressive, commonly EBV-containing neoplasms, and are treated with chemotherapy and/or radiotherapy.

More notably, a newly described entity, named fibrin-associated large B-cell lymphoma (FA-LBCL) was included in the WHO classification as a distinct type of DLBCL-CI with a highly favorable clinical outcome. It has been reported in association with cardiac prostheses, walls of pseudocysts, and cardiac myxomas, and characteristically does not show effacement of underlying tissue. In a recent study of 12 cases [6,7], the authors identified type III EBV latency in all tested cases, as seen in DLBCL-CI.

We have summarized the differences between FA-LBCL and DLBCl-CI in Table 1.

The present case most closely resembles what has been described as FA-LBCL. Our patient’s lymphoma was diagnosed post-operatively; pre-operative diagnosis or suspicion would have been extremely unlikely in this clinical context. The symptoms and imaging were suggestive of thrombosis, a far more common condition that would not warrant a biopsy. This patient’s staging evaluation and subsequent treatment could only happen after surgical excision and pathologic evaluation.

The decision-making process for post-surgical management is based on accurate distinction between FA-LBCL and other EBV-associated large B-cell lymphomas. Key histopathologic criteria include localization within fibrin, necrotic debris, or acellular exudate, strong EBV (EBER) positivity, absence of parenchymal infiltration or mass formation, and lack of significant inflammatory background. The next step includes imaging studies to exclude systemic disease. This is followed by surgical management that involves complete excision of the fibrin-associated lesion (e.g., cyst wall, prosthetic capsule, or thrombus) and removal of any foreign material.

In patients requiring systemic chemotherapy, the recommended interval between surgery and initiation of chemotherapy is 3–4 weeks for wound healing since early initiation (<2 weeks) may increase postoperative infection risk due to rituximab- and steroid-induced immunosuppression. It also allows time for definitive histopathologic review and staging results and ensures recovery of organ function before cytotoxic exposure. This delay is acceptable since FA-LBCL’s indolent nature allows a short delay without risk of progression.

## 4. Conclusions

In summary, we described here a case of a localized cardiac prosthesis fibrin-associated EBV-positive diffuse large B-cell lymphoma, with no evidence of progression or recurrence after 59 months of follow-up. More studies, including long-term follow-up to clarify the most appropriate management for this group of patients, who usually have significant cardiac comorbidity, are needed.

## 5. Learning Points

- FA-LBCL is an indolent EBV-positive lymphoma confined to fibrin.
  - It typically arises in acellular fibrin or necrotic material associated with pseudocysts, prostheses, or thrombi, without parenchymal invasion.
  - Recognition of this confined growth pattern is essential to avoid over-diagnosis as aggressive DLBCL.
- Histopathologic confirmation and radiologic correlation are crucial.
  - The diagnosis requires demonstration of EBV (EBER) positivity and exclusion of systemic or mass-forming disease on imaging.
  - Immunophenotype is typically CD20+, CD30 variable, and CD10/BCL6 negative, reflecting a non-germinal center B-cell phenotype.
- Surgical excision alone is curative in most patients.
  - Removal of the fibrinous or prosthetic nidus eliminates the immune-privileged environment driving EBV proliferation.
  - Adjuvant chemotherapy is unnecessary when complete excision is achieved, and disease remains localized.
- Systemic therapy is reserved for select cases.
  - R-CHOP or rituximab monotherapy may be considered only for incomplete excision, multifocal lesions, or disseminated disease.
  - When systemic therapy is indicated, initiation should be delayed 3–4 weeks postoperatively to allow adequate wound healing.
- Follow-up should be conservative but structured.
  - Periodic clinical and imaging surveillance (every 6–12 months) is appropriate.
  - Recurrence is exceedingly rare, but long-term follow-up helps confirm durable remission.
- Key distinction from DLBCL associated with chronic inflammation (DLBCL-CI).
  - FA-LBCL is non-mass-forming, localized, and curable with surgery alone.
  - DLBCL-CI, in contrast, is mass-forming, destructive, and requires systemic chemoimmunotherapy.

## Figures and Tables

**Table 1:** Comparison of fibrin-associated large B-cell lymphoma (FA-LBCL) and DLBCL associated with chronic inflammation (DLBCL-CI).

	FA-LBCL	(DLBCL-CI)
Definition	EBV-positive large B-cell neoplasm confined to fibrin or necrotic material without tissue invasion	EBV-positive DLBCL developing in a setting of long-standing chronic inflammation, typically forming a mass lesion
Typical Site	Fibrinous or necrotic spaces: pseudocysts, chronic hematomas, prosthetic capsules, cardiac thrombi	Long-standing pyothorax (most classic), chronic osteomyelitis, metallic implants, chronic ulcers
Macroscopic Appearance	No discrete mass; often incidental finding within fibrin or cyst wall	Mass-forming lesion with surrounding fibrosis and inflammatory reaction
Histology	Large, atypical B-cells scattered within fibrin; no tissue infiltration	Sheets of large, atypical B-cells infiltrating tissue with necrosis and inflammatory background
EBV Status	Uniformly EBV-positive (EBER+)	EBV-positive in majority (>90%)
Host Immune Status	Usually immunocompetent	Often occurs in localized immune-privileged environment (chronic inflammation)
Tumor Microenvironment	Acellular fibrin matrix, no significant inflammation	Dense inflammatory infiltrate and fibrosis
Clinical Course	Indolent, localized; often cured by excision alone	Aggressive, locally destructive, may disseminate
Prognosis	Excellent (>95% disease-free with surgery alone)	Poorer prognosis; 5-year survival ~40–50% despite therapy
Standard Management	Complete surgical excision ± observation	Systemic chemoimmunotherapy (R-CHOP ± radiotherapy)
Recurrence/Progression	Extremely rare after complete excision	Common; relapse or progression frequent without therapy
WHO 2022 Classification	EBV-positive B-cell lymphoma, fibrin-associated type	EBV-positive DLBCL associated with chronic inflammation

## Data Availability

The original contributions presented in this study are included in the article. Further inquiries can be directed to the corresponding author.
